# Perforated Duodenal Diverticulitis: A Case Report of a Rare Surgical Entity Treated by Roux-en-Y Deriving Intestinal Patch

**DOI:** 10.7759/cureus.23167

**Published:** 2022-03-15

**Authors:** Evangelos Kalogiannis, Stefano Gussago, Dimitri Chappalley, Ian Fournier

**Affiliations:** 1 Division of Visceral Surgery, Department of Surgery, Sion Cantonal Hospital, Sion, CHE; 2 Department of Visceral Surgery and Transplantation, Geneva University Hospital, Geneva, CHE

**Keywords:** duodenal derivation, duodenal diverticulitis, intestinal patch, duodenal diverticulum, duodenal perforation

## Abstract

Perforated diverticulitis is a rare but serious complication associated with a significant mortality rate. Although many cases of conservative treatment have been reported, surgery remains the mainstay for perforated duodenal diverticulitis.

We report a rare case of a 55-year-old female who presented with epigastric pain without fever. Computed tomography revealed a 3 cm perforated duodenal diverticulum of the D2 part of the duodenum with a localized abscess. After the failure of conservative treatment, we performed a deriving intestinal patch completed by cholecystectomy and biliary decompression via a transcystic drain, as well as feeding jejunostomy. The patient was discharged on day 32. Removal of the transcystic drainage at eight weeks postoperatively was complicated by the appearance of an iatrogenic bilioperitoneum, which was effectively treated with percutaneous drainage.

Surgery remains challenging; our experience suggests that perforation covering with a deriving jejunal patch offers an alternative to direct beach suturing when the latter is deemed precarious. Part of the treatment success lies in local drainage and duodenal exclusion that can be achieved by various surgical approaches.

## Introduction

Following the colon, the duodenum is the second most common location of intestinal diverticula, with an incidence of duodenal diverticulum detected in autopsy between 3.3% to 22% [1]. The patients are usually asymptomatic, with only 10% developing symptoms, and 1%-2% needing eventually a surgical procedure [1]. There are two types of duodenal diverticulums, congenital and acquired. Congenital diverticula, being probably the result of a failed recanalization at seven to 10 weeks of gestation, located in the medial wall of the second and third portions of the duodenum, have a lower incidence, and contain all three layers of the duodenal wall. Acquired diverticula are principally pulsion diverticula in which the mucosa and submucosa herniate through a focal defect in the intestinal wall. The second and third duodenal portion is generally the most affected region in 58% and 26% of the cases, respectively [2]. Acquired diverticula may occur as sequelae of peptic disease or cholecystitis, with an endoscopic case series showing an increased prevalence of duodenal diverticulum in patients with cholelithiasis and choledocholithiasis, possibly because of the combined effects of biliary stasis and bacterial contamination [3].

Most diverticula are asymptomatic and only 10% of the patients experience complications such as acute diverticulitis, hemorrhage, perforation, biliary obstruction, or pancreatitis [4]. The main causes of perforated duodenal diverticulum can be diverticulitis, ulceration, enterolithiasis, foreign body, or blunt abdominal trauma, with diverticulitis reported to be the most common cause with a rate up to 62% [5]. A perforated duodenal diverticulum is a rare but serious complication associated with significant mortality reported up to 30% [6].

In current literature, more than 200 cases of perforated duodenal diverticulum have been reported since 1907: conservative treatment, primary closure, diverticulum resection, or combination of surgical and endoscopic drainage have been described but, still, no specific treatment has been proposed as the standard of care [7].

Herein, we report the rare case of a 55-year-old patient who presented with perforated duodenal diverticulitis requiring a surgical approach by deriving an intestinal patch after the failure of conservative treatment.

This article was previously presented as a meeting abstract at the 108th Annual Swiss Congress of Surgery on 1-3 June 2021.

## Case presentation

**Figure 1:**
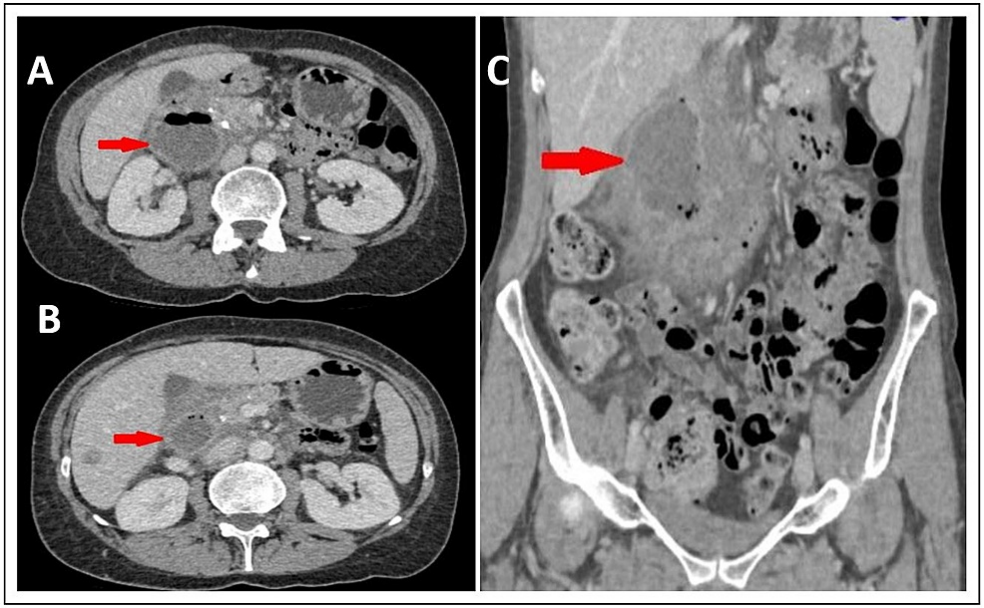
Preoperative CT scan Axial (A, B) and coronal (C) slides of the preoperative CT scan showing a D2 duodenal collection of 75 x 36 x 44 mm (red arrows)

A 55-year-old Caucasian female with unremarkable past medical history was addressed to our emergency department for acute epigastric pain radiating to the back. No associated nausea, vomiting, or fever was reported. Blood tests showed no significant leukocytosis (12G/L) with a normal level of C-reactive protein. Contrast-enhanced abdominal CT scan found a large duodenal diverticulum (3 cm) with digestive content and thickened walls associated with an infiltrated aspect of the peridiverticular fat. We initially opted for conservative treatment: an aspirative nasogastric tube was placed, antibiotic therapy with amoxicillin/clavulanic acid IV was started in association with proton pump inhibitors (PPIs) in therapeutic dosage (Esomeprazole 40 mg IV 1x/24h) and total parenteral nutrition was administered via central venous line. Radiological follow-up via contrast-enhanced abdominal CT scan at day 4 showed regression of the size of the perforation surrounding the duodenal diverticulum. The clinical and biological course was uneventful: a well-tolerated oral feeding was progressively started on day 7, IV antibiotic therapy was pursued until day 13 and the patient was dismissed on day 14 only with oral PPIs therapy (Esomeprazole 40 mg once daily). A day after discharge, on day 15, the patient consulted the emergency department of our hospital due to recurrence of epigastric and right hypochondrium pain. A new contrast-enhanced abdominal CT scan revealed a para-duodenal collection with solid debris arising from the D2 segment of the duodenum, measuring 68 x 30 x 33 mm. Conservative treatment with the previous protocol was reattempted but a follow-up CT scan at day 19 showed an increase in the size of the collection compared to the previous imaging (75 x 36 x 44 mm versus 68 x 30 x 33 mm respectively) (Figure 1).

Considering the unfavorable evolution, we decided to proceed with surgical treatment on day 21. Through a right Kocher incision, we entered the abdominal cavity and performed a Kocher maneuver in order to drain the retroperitoneal abscess. After mobilization of the duodenum from the vein cava to the renal vein and abundant lavage, we visualized a perforation of the posterior part of the duodenum, of a diameter of 2 cm, medially and inferiorly of the ampulla of Vater. We catheterized the perforation with a Foley catheter used as a guide for the eventual reparation. We then perform a “takedown” cholecystectomy followed by biliary drainage through a trans-cystic Escat drain. A methylene blue test through the cystic drainage assured the permeability of the common bile duct (CBD) and the absence of extra-biliary leakage.

**Figure 2:**
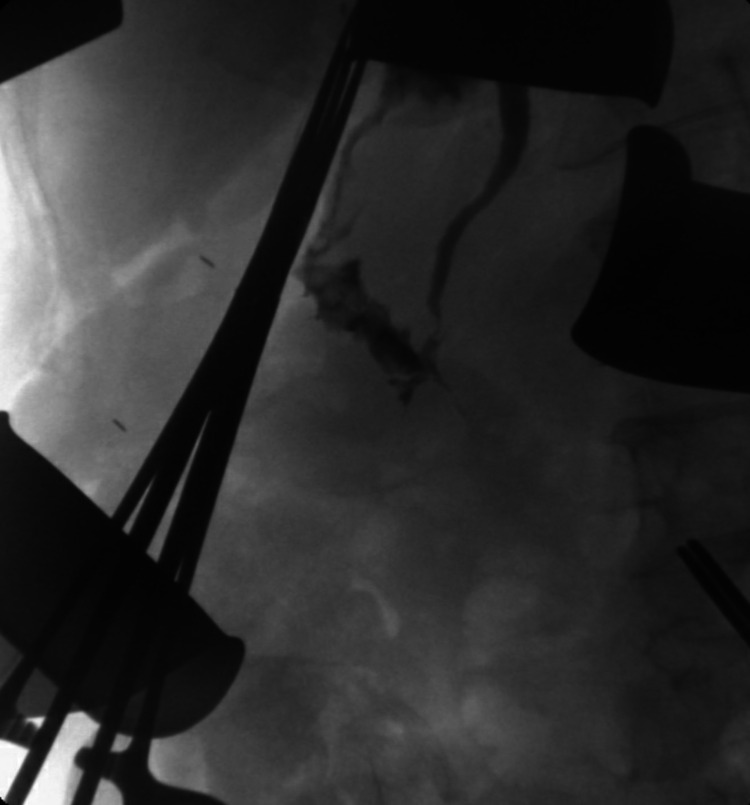
Perioperative cholangiography Postanastomotic cholangiography through the transcystic Escat drain attesting to the permeability of the common bile duct and the absence of anastomotic leakage

**Figure 3:**
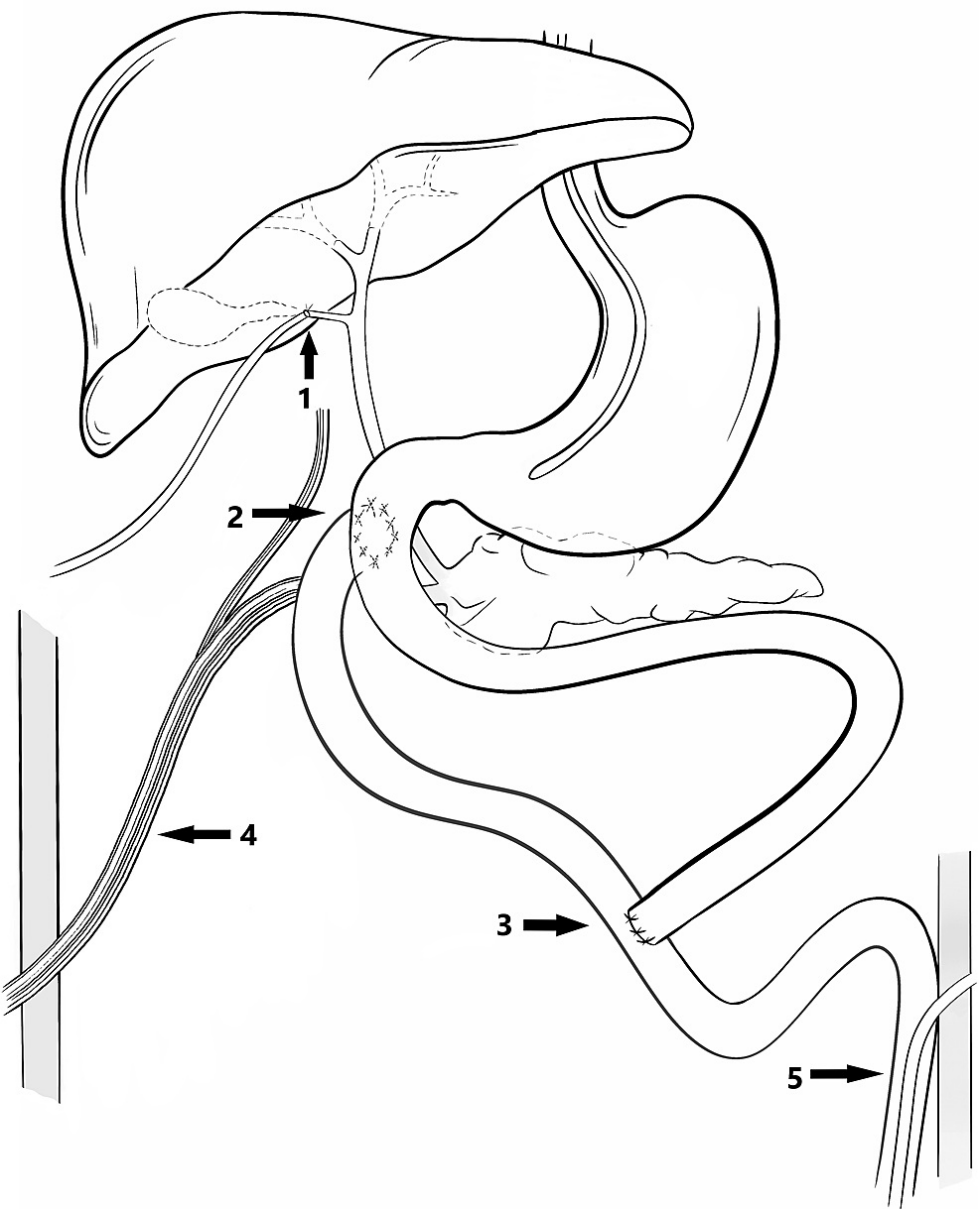
Surgical procedure 1. “Takedown” cholecystectomy followed by cholangiography through a transcystic Escat drain; 2. Duodenal repair with coverage by a trans-mesocolic intestinal Y-shaped patch; 3. Anastomosis of the foot of the loop end-to-side at 50 cm from the Treitz angle; 4. Drainage of Morrison’s space and the retroduodenal space with two multitube drainages; 5. Confection of a “Witzel” jejunostomy 15 cm from the foot of the loop anastomosis

We proceeded to a duodenal repair with coverage by a trans-mesocolic intestinal Y-shaped patch and anastomosis of the foot of the loop end-to-side at 50 cm from the Treitz angle. The Foley catheter was removed during the patching. Cholangiography through the trans-cystic Escat drain attested permeability of the CBD and absence of anastomotic leakage (Figure 2). We drained Morrison’s space and the retroduodenal space with two multitube drainages. Figure 2 shows the confection of a “Witzel” jejunostomy 15 cm from the foot of the loop anastomosis (Figure 3).

**Figure 4:**
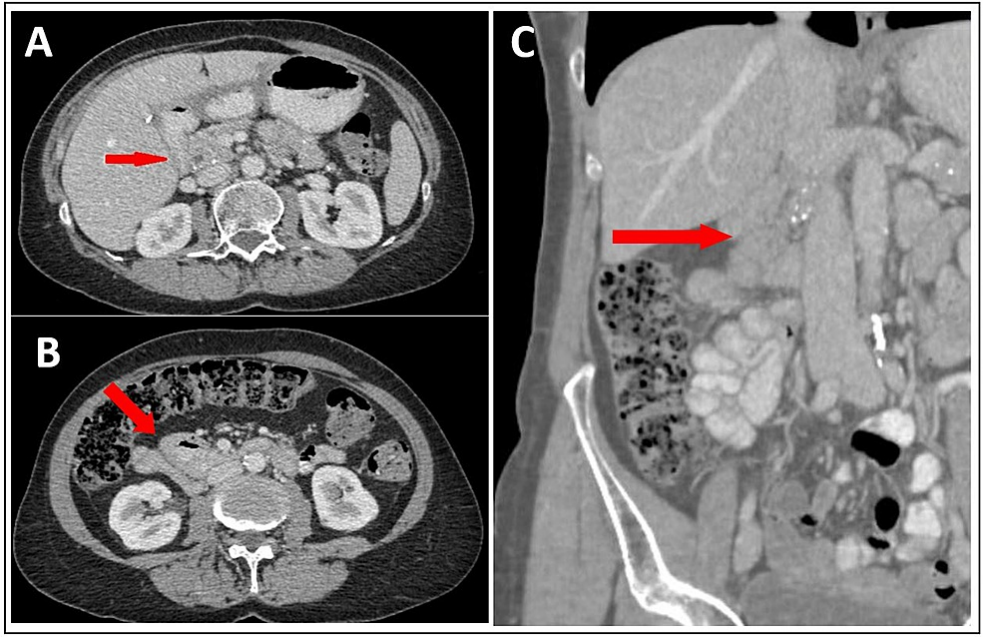
Postoperative CT scan Axial (A) and coronal (C) CT scan slides on postoperative day 13 showing no relapse of the abscess (red arrows) and no anastomotic leak (B, red arrow)

The intervention was uneventful. We modified the antibiotic therapy to piperacillin-tazobactam 4.5 g three times a day IV until postoperative day 7. Perioperative liquid cultures were positive for *Hafnia Alpei*, thus according to the infectiologists, no complementary antibiotic treatment was needed. Due to a drainage leak suspect either for fistula or infection on day 9, we administered a dose of octreotide intramuscularly (Sandostatin LAR 20 mg) and reintroduced the antibiotic treatment with piperacillin-tazobactam for a total of 27 days postoperatively leading to a favorable response. A follow-up contrast-enhanced abdominal CT scan on postoperative day 13 showed a significant regression of the collection (Figure 4). The transcystic drain was clamped at day 16 with a transitional increase of the liver test values, which are improved spontaneously on the following days. The postoperative course, otherwise uneventful, was complicated by malnutrition treated by parenteral nutrition, which was gradually weaned off thanks to progressive enteral refeeding by jejunostomy and progressive oral refeeding. A nasogastric tube was left in place until day 18. The patient was discharged on day 32 with the transcystic drain in place.

The transcystic drain was removed after eight weeks postoperatively. The patient subsequently developed an iatrogenic bilioperitoneum due to bile leakage from the cystic stump post-ablation of the Escat drain, treated by radiological puncture drainage. During the same hospitalization, the feeding jejunostomy was also removed since the patient had reached a satisfactory nutritional status and oral intake was covering the daily needs. The clinical evolution was favorable, and the patient was discharged after seven days.

A follow-up at four, six, and 12 months postoperatively confirmed a favorable clinical course and a significant improvement of the nutritional status.

## Discussion

Perforated duodenal diverticulitis is a rare though potentially life-threatening complication. In clinically stable patients and provided that close clinical monitoring is possible, the conservative treatment seems to be a safe approach, with the step-up approach to drainage (percutaneous or endoscopic) or even surgery in case of treatment failure [2].

The mainstay of conservative treatment is bowel rest with or without nasogastric tube suction associated with total parenteral nutrition and intravenous broad-spectrum antibiotics [8].

Percutaneous drainage can also be pursued as adjunct conservative treatment in some cases. Krishna et al. in a retrospective chart review with 14 cases with post-ERCP perforation (12 patients presented with duodenal and two with biliary perforation), seven patients (50%) underwent successful percutaneous drainage. However, percutaneous drainage was reserved for patients with localized fluid collections who did not present a major contrast leak or solid debris on a CT scan [9].

In clinically stable patients with a collection strictly limited to retroperitoneal space, endoscopic drainage of the perforation could be an alternative to percutaneous drainage [8]. Drain placement endoscopically through the diverticulum, endoscopic tissue shielding using polyglycolic acid sheets and fibrin glue, or endoscopic negative pressure therapy (ENPT) have been successful non-surgical treatments in a limited number of cases published as cases reports or cases series [7-8].

Recent literature shows a tendency towards a step-up approach. Kapp et al., in a systematic review, including a total of 47 patients with perforated duodenal diverticula from 2002 to 2020, showed that 16 patients (34%) were either treated conservatively (11/47) or initially treated conservatively with subsequent step-up to surgery (5/47) [2].

However, in case of clinical or radiological worsening, surgery must be pursued [9]. The most common surgical approach involves a stapled or hand-sewn diverticulectomy [8]. Nevertheless, surgery remains challenging due to the anatomical complexity of the duodenum, especially when it concerns the D2 part and the periampullary region. Furthermore, when the above perforation is associated with peridiverticular inflammation and fragility of the duodenal wall, diversion of the enteric flow by a digestive bypass is indicated in order to protect the diverticulectomy and reduce the risk of duodenal leak or fistula [10]. Many bypass surgical procedures have been described, varying from Roux-en-Y duodenojejunostomy with gastro-jejunal exclusion to truncal vagotomy/antrectomy with Billroth II gastrojejunostomy or even pancreaticoduodenectomy in two cases of complex perforated D2 diverticula [10-13].

No matter the type of surgical intervention, special attention should be given to the location of the diverticulum in relation to the biliary system. In order to achieve this, it is recommended to insert a Fogarty balloon catheter into the duodenum through a choledochotomy or cholecystotomy, thereby allowing the surgeon to safely identify the ampulla of Vater [14]. In addition, for the purpose of avoiding biliary leakage, external drainage with or without ERCP, papillotomy, and placement of a biliary stent are also recommended [2].

In our case, considering the initial favorable outcome under conservative treatment with nasogastric tube suction associated with total parenteral nutrition and intravenous broad-spectrum antibiotics, we decide to attempt a second conservative treatment. However, in light of an unfavorable radiological evolution on day 5 of the second conservative treatment, and presentation with an increase of the retroperitoneal abscess containing solid debris, we leaned in favor of surgical treatment.

The perioperative findings of a D2 periampullary diverticulum perforation 2 cm from the ampulla of Vater led us to rule out a diverticulectomy and decide to perform more invasive surgery. Hence, we were left with two surgical options: deriving Roux-en-Y jejunal patch versus pancreatoduodenectomy. In view of the patient’s young age and a rate of 23% of serious postoperative morbidities (Clavien-Dindo grade of at least 3) associated with pancreatoduodenectomy, we chose to perform a deriving jejunal patch [15].

Because of the localization of the diverticulum on the posterior D2 part of the duodenum, we proceeded to cholecystectomy in order to gain access to the CBD and evaluate directly the integrity of the papilla and its relation to the diverticulum. We decided to leave a trans-cystic Escat drain mainly for two reasons: on the one hand, to create a biliary diversion during the first postoperative days in order to reduce the amount of secretion on the freshly sewn anastomosis and thus avoid bile leakage; on the other hand, to protect the CBD in case of scarring stenosis occurring during the healing process due to the severe tissular inflammation linked to the chronic abscess.

With regard to our case, considering the proximity of the perforation to the ampulla of Vater in case of an inability to perform a Roux-en-Y jejunal patch, we were obliged to perform pancreatoduodenectomy. Fortunately, the intestinal patch was technically feasible and eventually successful. To the best of our knowledge, our case is the first to show the effectiveness of a Roux-en-Y jejunal patch for a D2, periampullary, complicated duodenal diverticulum perforation.

## Conclusions

Perforated duodenal diverticulitis is a rare, potentially life-threatening complication. Although no guidelines are currently available, a step-up approach is suggested by several authors. In our case, a step-up approach was attempted, with failure of the conservative treatment leading to a successful surgical intervention by Roux-en-Y deriving intestinal patch. Part of the treatment success lies in local drainage and digestive bypass that can be achieved by various surgical approaches. In our experience, this approach is a feasible and risk-acceptable technique when facing moderate to severe inflammation associated with periampullary perforated diverticulitis. This might allow avoiding, when possible, more complicated and invasive techniques such as duodenopancreatectomy in our case.
